# Impact of Functional Therapy on Skeletal Structures and Airways in Patients with Class II Malocclusion: Comparison of Treatment in Prepubertal and Pubertal Phases

**DOI:** 10.3390/life15071144

**Published:** 2025-07-20

**Authors:** Gianna Dipalma, Grazia Marinelli, Paola Bassi, Rosalba Lagioia, Francesca Calò, Mirka Cavino, Francesco Inchingolo, Franceska Vinjolli, Ioana Roxana Bordea, Giuseppe Minervini, Sabina Saccomanno, Andrea Palermo, Cinzia Maria Norma Maspero, Alessio Danilo Inchingolo, Angelo Michele Inchingolo

**Affiliations:** 1 Department of Interdisciplinary Medicine, University of Bari Aldo Moro, 70124 Bari, Italy; gianna.dipalma@uniba.it (G.D.); graziamarinelli@live.it (G.M.); paola.bassi@uniba.it (P.B.); rosalba.lagioia@uniba.it (R.L.); francesca.calo@uniba.it (F.C.); mirka.cavino@uniba.it (M.C.); francesco.inchingolo@uniba.it (F.I.); alessiodanilo.inchingolo@uniba.it (A.D.I.); or angelo.inchingolo@unimi.it (A.M.I.); 2 Department of Medical Sciences, Università Cattolica Nostra Signora del Buon Consiglio, Via Dritan Hoxha, 1001 Tirana, Albania; f.vinjolli@unizkm.al; 3 Department of Oral Rehabilitation, Faculty of Dentistry, Iuliu Hațieganu University of Medicine and Pharmacy, 400012 Cluj-Napoca, Romania; 4 Multidisciplinary Department of Medical-Surgical and Odontostomatological Specialties, University of Campania Luigi Vanvitelli, 80121 Naples, Italy; giuseppe.minervini@unicampania.it; 5 Department of Life, Health and Environmental Sciences, University of L’Aquila, 67100 L’Aquila, Italy; sabinasaccomanno@hotmail.it; 6 Department of Experimental Medicine, University of Salento, 73100 Lecce, Italy; andrea.palermo@unisalento.it; 7 Department of Biomedical, Surgical and Dental Sciences, University of Milan, 20100 Milan, Italy; cinzia.maspero@unimi.it; 8 Fondazione IRCCS Cà Granda, Ospedale Maggiore Policlinico, 20100 Milan, Italy

**Keywords:** class II malocclusion, Twin Block appliance, mandibular retrusion, skeletal maturation, upper airway, functional orthodontics

## Abstract

This study aimed to assess skeletal and upper airway modifications induced by the Twin Block appliance in patients with Class II malocclusion due to mandibular retrusion, using two-dimensional imaging techniques. A total of 11 patients (6 males, 5 females) were included and stratified into two groups (Pre-Peak and Peak) based on skeletal maturity evaluated through the cervical vertebral maturation (CVM) method. Lateral cephalometric radiographs were obtained at the beginning (T0) and end (T1) of treatment and analyzed using DeltaDent software. The appliance was worn full-time, except during meals and oral hygiene procedures, with monthly follow-ups. Significant changes were observed between T0 and T1 across the sample. Comparison between groups revealed statistically significant differences only in the H-C3a1 and vertical height (th) parameters (*p* < 0.05). In conclusion, the Twin Block appliance proved effective in enhancing mandibular and dental positioning, while also exerting a favorable influence on upper airway development.

## 1. Introduction

Class II malocclusion is one of the most frequently diagnosed skeletal-dental alterations in orthodontics [1,2,3]. It presents as a sagittal discrepancy between the dental arches, often due to a retruded mandible relative to the maxilla [4,5,6]. This condition can be skeletal, dentoalveolar, or mixed in origin, but in most cases, mandibular skeletal retrusion is predominant [7,8,9]. In addition to negatively impacting the facial profile—often perceived by patients as disharmonious—it may also be associated with functional disorders of the temporomandibular joint, mastication, and, in some cases, respiration [10,11,12,13].

Epidemiologically, Class II malocclusion is highly prevalent in the pediatric population, making it one of the primary challenges in interceptive orthodontics. Intervening during growth allows for the effective use of the patient’s skeletal development potential, achieving outcomes that are difficult to replicate in adulthood without the aid of orthognathic surgery [14,15,16,17].

The multifactorial etiology of Class II malocclusion requires thorough clinical and cephalometric evaluation to identify candidates for functional orthopedic treatment. In cases of evident mandibular retrusion with good remaining growth potential, functional appliances are an elective therapeutic option [18,19]. These devices are designed to stimulate mandibular growth and guide its development in an anteroinferior direction by harnessing the forces generated by orofacial musculature and altering mandibular posture [20,21,22].

Among the various options available, the Twin Block has established itself as one of the most effective and versatile functional appliances [23]. Designed by William J. Clark in the 1980s, it consists of two removable plates with inclined occlusal planes that guide the mandible forward during occlusion [24,25,26]. Its ease of use, customizability, and high patient compliance make it a common choice in daily clinical practice [27,28,29,30]. The Twin Block works according to a well-defined biomechanical principle: forced mandibular advancement stimulates condylar activity and promotes remodeling of the temporomandibular joint, encouraging skeletal growth in the sagittal direction [31,32,33]. At the same time, improvements in occlusal and soft tissue relationships are observed, resulting in a better facial profile and improved perioral aesthetics [34,35,36]. The recent literature has shown increasing interest in the potential impact of mandibular retrognathia on respiratory function, particularly in relation to obstructive sleep apnea syndrome (OSAS) [37,38,39]. A correlation has been observed between mandibular retrognathia and OSAS, a condition in which the upper airway is narrowed, particularly at the oropharyngeal level. Mandibular advancement induced by functional appliances such as the Twin Block can contribute to the enlargement of the pharyngeal airway, reducing resistance to airflow and improving nocturnal breathing quality [40,41,42]. However, despite the growing body of literature on functional appliances, two significant gaps remain. First, many studies fail to distinguish between prepubertal and pubertal growth phases, even though skeletal maturity plays a crucial role in treatment response [43,44,45]. Second, only a limited number of studies combine skeletal assessments with a systematic evaluation of upper airway dimensions, which may offer valuable insights into the broader functional effects of orthopedic treatment [46,47,48].

Understanding how skeletal and respiratory structures respond when treatment is initiated at different maturational stages is essential for improving clinical strategies. By focusing on two specific growth stages—prepubertal (CS1–CS2) and pubertal (CS3)—this study aims to contribute to a more comprehensive understanding of the craniofacial and airway changes associated with Twin Block therapy.

Therefore, the aim of this study is to evaluate the skeletal and upper airway changes induced by Twin Block therapy in patients with skeletal Class II malocclusion, comparing outcomes observed in individuals treated during the prepubertal phase (CS1–CS2) versus those treated during the pubertal growth peak (CS3), as assessed by the cervical vertebral maturation method.

## 2. Materials and Methods

### 2.1. Ethical Considerations

The study was conducted in compliance with the ethical principles outlined in the Declaration of Helsinki. All clinical data collected, including those deriving from cephalometric analyses, were obtained and processed in a compliant manner. Approval of the research protocol was obtained from the Ethics Committee “clinical study reference number 7593” to Prof. F. Inchingolo, U.O. of Odontostomatology. Prot. n°0015257|15/02/2023 AOUCPG23 COMET|P.

### 2.2. Sample and Study Design

The study included a sample of 11 growing patients (6 males and 5 females) with Class II skeletal malocclusion of mandibular origin, treated with functional Twin Block braces. All subjects were in good general health, without previous orthodontic treatment and with complete radiographic documentation before (T0) and after (T1) therapy. 

The inclusion criteria were as follows: skeletal Class II malocclusion, CVM stage CS1–CS3 at T1, age between 8 and 13 years, and no prior orthodontic treatment. 

Exclusion criteria included craniofacial syndromes, systemic diseases, and non-compliance during treatment.

Participants were divided into two groups based on the stage of skeletal maturation determined by the Cervical Vertebral Maturation (CVM) index, assessed on latero-lateral teleradiographs obtained at T0. This classification was based on a morphological analysis of cervical vertebrae C2, C3, and C4, considering shape of the vertebral body and presence of concavity on the lower edge.

Specifically, the groups were defined as follows:

- Prepubertal Group (CVM CS1–CS2): Patients in the early growth stage.

- Pubertal Group (CVM CS3): Patients approaching the pubertal growth peak stage.

The sample included 6 patients in the prepubertal group (CS1–CS2) and 5 patients in the pubertal group (CS3).

All treatments were performed by two experienced orthodontists, following a shared and standardized clinical protocol to ensure homogeneity of treatment procedures.

All appliances were fabricated by the same laboratory using a standardized construction bite protocol, ensuring consistency in device design.

### 2.3. Treatment Protocol

The device used was the Twin Block, built according to Clark’s specifications. Patients were instructed to wear the device for 24 h a day, removing it only during meals and oral hygiene procedures. Clinical checks were performed at regular intervals of about 4 weeks. During each visit, the anteroposterior relationship between the arches was assessed, with and without braces in place. Active treatment was considered complete when the mandible maintained a stable advanced position even in the absence of the device. Next, the Twin Block was replaced with an upper plate equipped with a mandibular protrusion segment in order to guide the correct eruption of the premolars. Treatment was completed when a Class I molar-to-canine ratio was achieved.

### 2.4. Cephalometric Analysis

Cephalometric analysis was conducted by a single calibrated examiner to ensure consistency and eliminate inter-operator variability. The examiner was blinded to group allocation to reduce assessment bias.

Latero-lateral teleradiographs in standard projection were acquired pre-treatment (T0) and post-treatment (T1) and processed using DeltaDent cephalometric software. A single experienced operator performed all tracings and measurements, ensuring uniformity in evaluation.

The cephalometric points considered are shown in Table 1, which illustrates the references used for tracing. Upper airway measurements were made by drawing lines parallel to the Frankfurt (Po-Or) plane, identifying posterior pharyngeal references at the ve, p, ph, and eb points.

### 2.5. Statistical Analysis

Intra-operator error was evaluated by repeating the tracings on 5 subjects randomly selected from the sample. A paired samples *t*-test was applied to compare the replicate measurements to assess the reproducibility of the method.

Statistical analysis of the data was performed using IBM SPSS Statistics v.20, M-DEAP v.2, and Microsoft Excel 365 software. The *t*-test was used to compare pre- and post-treatment cephalometric values. Changes were considered statistically significant for *p* < 0.05.

## 3. Results

### 3.1. Results for the Whole Sample

The treatment goal was achieved in all subjects included in the study, with complete correction of Class II malocclusion and achievement of a Class I molar relation.

As described, cephalometric measurements taken at baseline (T0) and at the end of treatment (T1) on the entire sample (n = 11) were analyzed. The averages of the measurements for each time point and the changes (Δ = T1 − T0) were calculated and analyzed for statistical significance using the *t*-test for paired data.

Statistical analysis revealed significant changes in several cephalometric variables. In particular, measurements such as S-Pns, p-pp, SNB, Co-Gn, Overjet, Overbite, N-Me, Ans-Me, Length (tt-eb), and Height (th) showed statistically significant changes (*p* < 0.05), indicating positive effects of the Twin Block device not only on mandibular position and dento-skeletal relationships but also on upper airway dimensions.

### 3.2. Results by Growth Stage Subgroups

The sample was divided into two subgroups: the Pre-Peak group (CS1–CS2) and the Peak group (CS3). For each group, the averages of measurements at T0 and T1, changes, and relative *p*-values were reported in order to assess the influence of growth stage on functional orthopedic treatment.

The results indicate differences between the two subgroups. In the Peak group, which is characterized by greater skeletal maturation, more pronounced average increases in nasopharynx and oropharynx dimensions were observed, as evidenced by S-Pns, ad1-Pns, and AA-Pns values. Soft palate (Ans^Pns^P) and hypopharynx (eb-peb) measurements also showed improvements in the Peak group, suggesting a greater impact of treatment in the presence of active growth.

In contrast, in the Pre-Peak group, mandibular variables such as SNB and Co-Gn showed more pronounced changes, indicating a more pronounced effect on mandibular growth when treatment is undertaken in the early stages of skeletal development.

Regarding facial heights (N-Me and Ans-Me), greater increases were observed in the Peak group, suggesting a more harmonious craniofacial response in the presence of pubertal drive. Measurements of tongue position and size (Length tt-eb) and the hyoid bone (H-C3a1) showed statistically significant changes in the Peak group, including an increase in lingual length and caudal displacement of the hyoid bone.

Finally, the only statistically significant difference between the two groups was in the variables Height (th) (*p* = 0.02) and H—C3a1 (*p* = 0.03), suggesting greater responsiveness of parapharyngeal structures in the peak growth phase subjects.

Overall, the data indicate that Twin Block treatment produces favorable effects on craniofacial and upper airway structures, with variations being influenced by the stage of skeletal maturation assessed according to the CVM method. 

No correction for multiple comparisons was applied due to the exploratory nature of the analysis and small sample size.

## 4. Discussion

The relationship between craniofacial growth and airway development is well established, and this study aimed to provide new evidence on how these aspects evolve in patients treated with the Twin Block appliance at different skeletal maturation stages.

The findings confirm that functional orthopedic treatment can be effective not only in correcting sagittal skeletal discrepancies but also in promoting positive changes in upper airway morphology.

In the present study, the primary objective was to evaluate the effect of Twin Block functional appliance treatment on upper airway dimensions and mandibular growth in patients with Class II malocclusion [49,50,51,52]. The results confirmed a significant positive impact, both in terms of improved mandibular position, as evidenced by increases in Co-Gn measurements, and in increased upper airway size, detected by measurements such as S-Pns and p-pp [53,54,55,56,57]. These results are consistent with previous studies, such as that of Vinoth et al., which observed significant increases in mandibular branch length and airway extension in children treated with Twin Block [58,59,60,61].

Subsequently, stratification of patients according to the degree of cervical vertebral maturation (CVM), based on Lamparski’s method, allowed us to analyze the influence of treatment timing on cephalometric outcomes [62,63,64]. Comparison between the Pre-Peak (CS1 and CS2) and Peak (CS3) groups revealed that while both groups showed improvements in mandibular and airway dimensions, the Peak group exhibited more pronounced increases in some measurements, such as those related to the nasopharynx (S-Pns, ad1-Pns), soft palate (Ans^Pns^P), and hypopharynx (eb-peb). In contrast, mandibular SNB and Co-Gn parameters showed more pronounced improvements in the Pre-Peak group, suggesting that mandibular growth stimulation may be more effective when treatment begins in earlier skeletal phases. These data are in line with the findings of Khoja et al., who observed that the Twin Block appliance best exploits the pubertal growth peak to maximize skeletal effects, reducing the overall treatment duration. Perinetti et al. also confirmed the effectiveness of functional treatment during puberty, emphasizing how timing is a key factor in achieving lasting and significant results [65,66,67]. Although not all airway parameters reached statistical significance, the general trend toward improved airway patency suggests a potential reduction in respiratory resistance and possible benefits for patients with functional breathing disorders such as obstructive sleep apnea. Of particular note is the significant increase in tongue length (Height (th)) and concomitant displacement of the hyoid bone (H-C3a1) in the Peak group [68,69,70]. These changes may indicate functional adaptation of the oropharyngeal region following mandibular advancement. In clinical terms, such anatomical modifications may contribute to increased airway patency and potentially improve airflow during respiration, particularly during sleep. In conclusion, our results indicate that both prepubertal and pubertal phases elicit distinct skeletal and airway responses to Twin Block therapy. While early treatment may better stimulate mandibular growth, pubertal treatment appears more effective in expanding airway dimensions and inducing soft tissue adaptations.

These findings support the need for individualized treatment planning based on skeletal maturation and clinical objectives, without establishing rigid recommendations for treatment timing.

Despite these promising findings, certain limitations of the study must be acknowledged.

### Limitations

We acknowledge that the limited sample size (n = 11) represents a significant limitation of this study. However, the selection of participants was intentionally rigorous in order to include only patients who were either in the prepubertal (CS1–CS2) or pubertal (CS3) growth phases, as determined by cervical vertebral maturation. This strict inclusion criterion was necessary to ensure homogeneous subgroups and enhance the reliability of growth phase comparisons. Moreover, the study required a long observational period, since data collection was completed only after each patient had finished the full course of functional treatment. While this careful approach increased the internal validity of the findings, it also limited the number of eligible subjects.

Despite this limitation, the analysis revealed significant skeletal and airway modifications, providing valuable preliminary insights. For this reason, we considered it important to publish these first results to offer a contribution to the scientific community and stimulate further research in this specific area of orthodontics.

We are fully aware of the need for future studies with larger cohorts and more robust statistical power, including formal power analysis, to confirm and expand upon these preliminary results.

## 5. Conclusions

Treatment of Class II malocclusion with the Twin Block appliance has been confirmed to be effective not only in improving tooth and mandibular position but also in promoting a significant increase in upper airway size. This finding is of particular importance for patients with respiratory problems associated with craniofacial dysfunction and malocclusion.

This study confirmed that treatment of skeletal Class II malocclusion with the Twin Block appliance can produce significant skeletal and airway improvements. Specifically, patients treated during the prepubertal phase showed more pronounced mandibular growth changes, while those treated during the pubertal growth peak exhibited greater increases in upper airway dimensions and soft tissue adaptations. Although limited by the small sample size, these findings suggest that skeletal maturation stage influences the nature of treatment response. Future studies on larger cohorts are necessary to validate these preliminary observations and support clinical decision-making based on individual growth phases.

These findings should be interpreted with caution and validated by future longitudinal research on larger cohorts, using three-dimensional imaging and functional airway assessment.

## Figures and Tables

**Table 1:** A diagram of all cephalometric points used.

Measure	Definition
Nosepharynx	
S-PNS	Distance from the saddle (S) to the posterior nasal spine (PNS).
AD1-PNS	Distance from AD1 to posterior nasal spine (PNS). AD1 is the point of intersection of the posterior pharyngeal wall with the line joining posterior nasal spine (PNS) and basion (Ba).
AD2-PNS	Distance from AD2 to the posterior nasal spine (PNS). AD2 is the point of intersection of the posterior pharyngeal wall with the line from the midpoint of the line from the saddle (S) to the basion (Ba) to the posterior nasal spine (PNS).
Oropharynx	
AA-PNS	Distance from the most anterior point of the atlas (AA) to the posterior nasal spine (PNS).
VE-PVE	Distance from the soft palate point closest to the posterior pharyngeal wall (velum palatinum, VE) to the corresponding horizontal point on the posterior pharyngeal wall (PVE).
P-PP	Distance from the tip of the soft palate (P) to the corresponding horizontal point on the wall posterior pharyngeal (PP).
PAS	Distance from the points of intersection on the anterior and posterior pharyngeal wall to the line joining the supramental (B) to the gonion (Go).
PH-PPH	Distance of the corresponding horizontal points on the anterior and posterior pharyngeal wall at level of the oropharynx in its narrowest area.
Soft Palate	
ANS-PNS-P	Angle from anterior nasal spine (ANS) to posterior nasal spine (PNS) to palatal point (P).
PNS-P	Distance from the posterior nasal spine (PNS) to the tip of the soft palate (P).
SP1-SP2	Cross section of the thickest part of the soft palate.
Hypopharynx	
EB-PEB	Distance from the vallecula of the epiglottis (EB) to the corresponding horizontal point on the posterior pharyngeal wall (PEB).
Maxilla	
SNA	Angle from saddle (S) to nasion (N) to subspinal (A).
ANS-PNS	Palatal plane length from anterior nasal spine (ANS) to posterior nasal spine (PNS).
Jaw	
SNB	Angle from saddle (S) to nasion (N) to supramental (B).
ANB	Angle from subspinal (A) to nasion (N) to supramental (B).
NS-MP	Angle from nasion (N) to saddle (S) to mandibular plane (MP). The mandibular plane is the line joining the point of the mandibular base (MBP) to the chin (ME).
CO-GN	Mandibular length. The length from the most posterior and superior point of the head condylar (CO) to the most anterior and inferior point of the mandibular symphysis (GN).
C3AI-HPT-RGN	Sum of two distances: (1) the perpendicular distance between the most anterior and lowest point of the body of the third cervical vertebra (C3AI) and HPT. HPT is the vertical line from the most anterior and superior point of the hyoid bone perpendicular to the line from the nasion (N) to the saddle (S) with an upward correction of 7°. (2) The distance from the most posterior point of the mandibular symphysis (retrognation, RGN) perpendicular to HPT.
Facial Heights	
N-ME	Distance from nasion (N) to chin (ME).
ANS-ME	Distance from anterior nasal spine (ANS) to chin (ME).
Tongue	
LENGTH (TT-EB)	Tongue length. The distance from the anterior point of the tip of the tongue (TT) to the base of the epiglottis (EB).
HEIGHT (TH)	Tongue height. The perpendicular distance from the highest point of the tongue (TH) located below the posterior nasal spine (PNS) to the line joining the tip of the tongue (TT) to the point of intersection of the tongue and mandibular border (TG).
Hyoid Bone	
H-H’	Distance from the most anterior and superior point of the hyoid bone (H) perpendicular to the plane mandibular (MP).
H-C3AI1	Hyoid (H). The perpendicular distance from the most anterior and superior point of the bone hyoid to the perpendicular line joining C3AI to HPT.

## Data Availability

The data are contained within the article.

## Abbreviations

Abbreviation	Definition
T0	Beginning of treatment
T1	End of treatment
CVM	Cervical Vertebral Maturation
SNB	Sella-Nasion-B point
Co-Gn	Condylion-Gnation
N-Me	Nasion-Menton
Ans-Me	Anterior Nasal Spine-Menton
S-Pns	Sella-Posterior Nasal Spine
p-pp	Soft palate lenght
tt-eb	Tongue tip to epiglottis base
th	Tongue height
H-C3a1	Hyoid bone to C3 anterior point
Ad1-Pns	Adenoid 1 to Posterior nasal spine
AA-Pns	Anterior atlas to Posterior nasal spine
Ans^Pns^P	Soft palate angulation
eb-peb	epiglottis base to posterior epiglottis base
CS1-CS3	Cervical stage 1 to 3
IBM SPSS	International business Machines Statistical Package for the social sciences
M-DEAP	Measurement and data evaluation analysis program
DeltaDent	Dental Cephalometric analysis software
